# Constructing Positively Charged Thin-Film Nanocomposite Nanofiltration Membranes with Enhanced Performance

**DOI:** 10.3390/polym12112526

**Published:** 2020-10-29

**Authors:** Wenyao Shao, Chenran Liu, Tong Yu, Ying Xiong, Zhuan Hong, Quanling Xie

**Affiliations:** 1 Technology Innovation Center for Exploitation of Marine Biological Resources, Third Institute of Oceanography, Ministry of Natural Resources, Xiamen 361005, China; 18805921314@163.com (C.L.); yutongtx_1@163.com (T.Y.); zhong@tio.org.cn (Z.H.); 2 Department of Chemical and Biochemical Engineering, College of Chemistry and Chemical Engineering, Xiamen University, Xiamen 361005, China; wyshao@xmu.edu.cn (W.S.); 18805921314@163.com (C.L.); 3 Guangdong Provincial Key Laboratory of Soil and Groundwater Pollution Control, School of Environmental Science and Engineering, Southern University of Science and Technology, Shenzhen 518055, China; xiongy@sustech.edu.cn; 4 Fujian Collaborative Innovation Center for Exploitation and Utilization of Marine Biological Resources, Xiamen 361005, China; yutongtx_1@163.com (T.Y.); zhong@tio.org.cn (Z.H.)

**Keywords:** positively charged, nanofiltration, thin-film nanocomposite, graphene oxide, polyethyleneimine, high performance

## Abstract

Using polyethylenimine (PEI) as the aqueous reactive monomers, a positively charged thin-film nanocomposite (TFN) nanofiltration (NF) membrane with enhanced performance was developed by successfully incorporating graphene oxide (GO) into the active layer. The effects of GO concentrations on the surface roughness, water contact angle, water flux, salt rejection, heavy metal removals, antifouling property, and chlorine resistance of the TFN membranes were evaluated in depth. The addition of 20 ppm GO facilitated the formation of thin, smooth, and hydrophilic nanocomposite active layers. Thus, the TFN-PEI-GO-20 membrane showed the optimal water flux of 70.3 L·m^−2^·h^−1^ without a loss of salt rejection, which was 36.8% higher than the thin-film composite (TFC) blank membrane. More importantly, owing to the positively charged surfaces, both the TFC-PEI-blank and TFN-PEI-GO membranes exhibited excellent rejections toward various heavy metal ions including Zn^2+^, Cd^2+^, Cu^2+^, Ni^2+^, and Pb^2+^. Additionally, compared with the negatively charged polypiperazine amide NF membrane, both the TFC-PEI-blank and TFN-PEI-GO-20 membranes demonstrated superior antifouling performance toward the cationic surfactants and basic protein due to their hydrophilic, smooth, and positively charged surface. Moreover, the TFN-PEI-GO membranes presented the improved chlorine resistances with the increasing GO concentration.

## 1. Introduction

Nowadays, many industries such as mining [1], metallurgy [2], electroplating [3], and microelectronic manufacturing [4] discharge a large amount of industrial wastewater contaminated by heavy metals. It is becoming a huge environmental and public risk due to the persistent and high toxicity of heavy metals to human beings and the environment. Therefore, it is urgent to develop efficient and practical solutions to remove heavy metals from industrial wastewater.

In the past few decades, various methods have been applied to remove or reclaim heavy metals, including chemical precipitation [5], ion exchange [6], adsorption [7], electrolysis [8], and membrane separation. Among these methods, membrane separation is considered as the most promising technique, owing to its high efficiency, environmental friendliness, small footprint, modular design, and easy operation. The pressure-driven membrane separation processes for removing heavy metals include ultrafiltration (UF) [9,10,11,12], nanofiltration (NF) [13,14,15,16], and reverse osmosis (RO) [17,18]. UF has the advantages of the lower operation pressure, higher flux, and lower investment cost compared with NF and RO. Nevertheless, the pore size of UF is too large to efficiently reject heavy metals. RO can completely reject heavy metal ions, but it requires the high operation pressure accompanying the high investment and energy costs. NF, with a nominal molecular weight cut-off (MWCO) of 200 to 1000 Da and pore size between UF and RO, finds the niches that need high rejections of heavy metals under the relatively low operational costs, which is very feasible to remove heavy metals from wastewater. It is well known that both size exclusion and Donnan exclusion are important separation mechanisms for NF process. That is to say, besides the surface pore size, the surface charge property of NF membrane also plays a crucial role in rejecting heavy metal ions.

Currently, most of the commercially available NF membranes are polyamides (PA) thin-film composite (TFC) membranes fabricated through interfacial polymerization (IP) between piperazine (PIP) and trimesoyl chloride (TMC) [19,20,21]. The PA TFC membranes are negatively charged because of the presence of abundant carboxyl groups derived from the hydrolysis of unreacted acyl chloride groups. The negatively charged PA NF membranes would decrease the effectiveness in the rejection of heavy metal ions due to the electrostatic attractions between the negatively charged surface and the positively charged heavy metal ions. A facile approach to improve cation rejection is to construct the positively charged NF membrane.

Polyethylenimine (PEI) is a known cationic polyelectrolyte with branched chain, containing primary amine groups, secondary amine groups, and tertiary amine groups [22]. PEI has been extensively studied to fabricate positively charged NF membranes because of its good hydrophilicity and high contents of amine groups [23,24,25]. Recently, many methods have been utilized to prepare PEI-based NF membranes, including IP [26,27,28], physical blending [29], layer-by-layer assembly [30,31], and surface grafting [32]. However, the long-term stabilities of the NF membranes fabricated via physical blending and layer-by-layer assembly are great challenges during the cross-flow membrane separation process, and the surface grafting method is too complex to industrialize. Compared with the other methods, the IP method is more attractive to construct the positively charged PEI-based PA NF membranes. However, the conventional PEI-based PA NF membrane via IP process still faces huge challenges for enhancing the permselectivity [33], antifouling ability [34], and chlorination resistance [35].

To overcome these challenges, novel thin-film nanocomposite (TFN) NF membranes have been extensively studied by incorporating various nanofillers into the active layer [36,37,38]. In recent years, two-dimensional graphene oxide (GO) [39,40,41,42] or its derivatives [43,44,45] having the unique structures and properties were successfully incorporated into the PIP-based PA NF membranes, which are helpful to significantly improve membrane separation. Although many studies have been done on the negatively charged PIP-based TFN NF membranes using GO nanofillers, little work has been done on the positively charged PEI-based TFN NF membranes.

In this study, in order to construct the positively charged NF membrane with enhanced performance, PEI and GO were used as the aqueous reactive monomers and the aqueous co-additives, respectively. The effects of GO concentrations on the resultant positively charged NF membranes were investigated in depth in terms of the surface roughness, water contact angle, water flux, salt rejection, heavy metal removals, antifouling property, and chlorine resistance. This novel, positively charged NF membrane demonstrated an exceptional removal of heavy metal ions with improved antifouling performance and chlorine resistance.

## 2. Experimental

### 2.1. Materials

Analytical grade piperazine (PIP, 99% purity), trimesoyl chloride (TMC, 98% purity), cetyl trimethyl ammonium chloride (CTAC, 97% purity), dodecyl trimethylammonium chloride (DTAC, 99% purity), PEI (*M*_w_ = 2000), and 4-dimethylaminopyridine (DMAP, 99% purity) were purchased from Shanghai Macklin Biochemical Company, Shanghai, China. Triethylamine (TEA, 99% purity), n-hexane, ethanol, sulfuric acid (H_2_SO_4_, 98% purity), hydrochloric acid (HCl, 37% purity), hydrogen peroxide (H_2_O_2_, 30% purity), sodium nitrate (NaNO_3_, 99.7% purity), sodium hypochlorite (NaClO, active chlorine content > 5.2%), sodium sulfate (Na_2_SO_4_, 99% purity), magnesium sulfate (MgSO_4_, 99% purity), sodium chloride (NaCl, 99.5% purity), magnesium chloride (MgCl_2_, 98% purity), copper chloride (CuCl_2_), nickel chloride (NiCl_2_), zinc chloride (ZnCl_2_), cadmium chloride (CdCl_2_), and lead nitrate (Pb(NO_3_)_2_) were purchased from Sinopharm Chemical Reagent Company, Shanghai, China. Lysozyme was obtained from Dingguo Changsheng Company, Beijing, China.

### 2.2. Preparation and Characterization of GO

According to our previous report [43], GO nanosheets were prepared through the modified Hummers method, and the resulting GO was further characterized by FT-IR (Bruker VERTXE 70, Fällanden, Switzerland), TEM (JEOL JEM 1400, Tokyo, Japan), XRD (Bruker D8 Discover, Karlsruhe, Germany), XPS (Physical Instruments Quantum 2000, Chanhassen, MN, USA), and Raman spectroscopy (Thermo Renishaw, West Dundee IL, IL, USA).

### 2.3. Preparation of NF Membranes

Polysulfone (PSU) UF membrane was lab-made and used as the supporting layer in our previous report [39]. The PSU support was fixed on a homemade polypropylene frame. GO was added into the aqueous solution consisting of 1.0 wt% PEI, 2.0 wt% TEA, and 0.08 wt% DMAP, which was treated by ultrasonic dispersion for 1 h. The obtained aqueous solution was poured on top of the PSU UF membrane and drained off after 1 h. Subsequently, 0.2 w/v% TMC organic solution was further poured on top of the PEI-saturated PSU support and removed after 4 min. Finally, the composite membrane was heated at 60 °C for 20 min. The obtained TFN membranes with incorporating GO were named TFN-PEI-GO-x, where x denotes the aqueous solution containing x ppm of GO in the aqueous solution. The control PEI-based TFC membrane using PEI monomers without addition of GO was named TFC-PEI-blank. The control PIP-based TFC membrane using PIP monomers without addition of GO in our previous study [43] was named TFC-PIP-blank. The membrane ID and the corresponding IP reaction conditions are listed in Table 1.

### 2.4. Characterization of NF Membranes

The cross-sectional and surface morphologies of the fabricated membranes were observed by SEM (LEO-1530, Jena, Germany). The surface roughness was recorded by atomic force microscopy (AFM, MI5500, Agilent, Santa Clara, CA, USA). The chemical groups of the membrane surfaces were characterized by attenuated total reflectance-Fourier transform infrared spectroscopy (ATR-FTIR, Bruker Vertex 70, Fällanden, Switzerland). The chemical compositions of the membrane surfaces were characterized by XPS (Physical Instruments Quantum 2000, Chanhassen, MN, USA). The membrane hydrophilicity was evaluated by a contact angle goniometer (Beijing HARKE SPCAX3, Beijing, China).

### 2.5. Performance of NF Membranes

The membrane separation performance was tested using a laboratory cross-flow membrane separation device with 70 cm^2^ effective area (FlowMem-0021-HP, FMT, Xiamen, China). The tested membranes were prepressured for 30 min under the transmembrane pressure of 0.5 MPa, the temperature of 25 °C, and the tangential velocity of 1.8 m/s. The salt rejections were first evaluated using four kinds of 2000 ppm salt solutions (Na_2_SO_4_, MgSO_4_, NaCl, and MgCl_2_). The removals of heavy metal ions were further evaluated using five kinds of 500 ppm heavy metal salt solutions (ZnCl_2_, CdCl_2_, CuCl_2_, NiCl_2_, and Pb(NO_3_)_2_). The salt rejections were measured under the full batch mode (both the concentration and permeate stream were recycled into the feed tank) to keep the feed concentration constant. The key parameters of the related ions are listed in Table 2.

The water flux and rejection were calculated by Equations (1) and (2), respectively:

(1) $$J=\frac{V}{A∆t}$$

where *J* is the water flux (L·m^−2^·h^−1^, abbreviated as LMH), V is the volume of the permeate (L), A is the effective area of the membrane (m^2^), and Δt is the filtration time (h).

(2) $$R\left(\%\right)=\left(1-\frac{C_{p}}{C_{c}}\right)\times 100\%$$

where *C_p_* and *C_c_* refer to the solute concentration in the permeate and in the concentrate, respectively. The salt concentration was calculated according to the standard curve between the concentration and the conductivity measured by a conductivity meter (Mettler Toledo S3, Schweiz, Switzerland).

Two kinds of cationic surfactants (CTAC, DTAC) and the basic protein (lysozyme) were used as the model foulants to evaluate the antifouling performance of the selected NF membranes. First, the pure water flux was recorded for 60 min at 0.5 MPa. Then, the pure water was replaced with a 2000 ppm solution containing the model foulant, and the flux of the pollutant solution was recorded for an additional 60 min. After water washing, both the water filtration procedure and pollutants’ filtration procedure were repeated.

After immersion in a 2000-ppm NaClO solution, the selected NF membranes were remeasured in terms of water flux and salt rejection. The chlorine resistances were evaluated based on the flux variation and the rejection variation.

## 3. Results and Discussion

### 3.1. Membrane Morphologies

**Figure 1:**
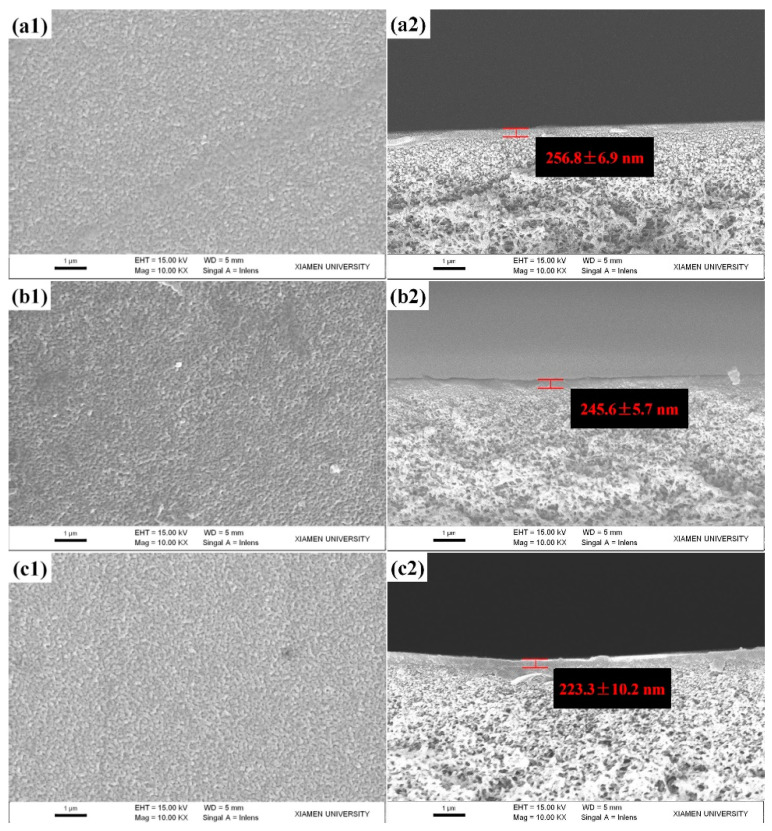
SEM images of top surfaces (column 1) and cross sections (column 2): (**a**) TFC-PEI-blank; (**b**) TFN-PEI-GO-20; (**c**) TFN-PEI-GO-40.

The surface and cross-section morphologies of the fabricated NF membranes were characterized by SEM, as shown in Figure 1. While using the small molecular PIP as aqueous reactive monomers, the TFC-PIP-blank membrane presented a dense surface with the typically discrete nodular structures [43]. While using the macromolecular PEI as aqueous reactive monomers, both the TFC-PEI (Figure 1(a1)) and TFN-PEI-GO membranes (Figure 1(b1,c1)) exhibited the dense and smooth surfaces without nodular structures. Although PEI had a large number of reactive groups (primary amine groups), its reactivity was still lower than PIP due to its large steric hindrance effect and low diffusion rate.

According to the cross-sectional SEM images (Figure 1(a2,b2,c2)), the thickness of the active layers decreased with the increasing GO concentration. The addition of GO into aqueous solution slowed the PEI diffusion toward the organic phase, resulting from the large steric hindrance of GO nanosheets and the hydrogen bonding interaction between GO and PEI. Namely, the IP reactivity between PEI and TMC was reduced by the introduced GO, which accordingly decreased the thickness of the active layer. The thickness of the active layer slightly decreased from 256.8 nm (TFC-PEI-blank, Figure 1(a2)) to 223.3 nm (TFN-PEI-GO-40, Figure 1(c2)). In this study, we used two approaches to promote more macromolecular PEI monomers to take part in the IP reaction. On the one hand, the impregnated time of aqueous solution was extended to 1 h and the IP reaction time was extended to 4 min. On the other hand, DMAP as an effective phase transfer catalyst was added into aqueous solutions to eliminate the steric hindrance of acyl transfer reaction and promote the IP reactions [47]. The introduction of DMAP into the aqueous solution significantly increased the degree of the cross-linking reaction between highly steric-hindered PEI and TMC [48]. As a result, both the TFC-PEI-blank and the TFN-PEI-GO membranes showed the thicker active layer compared with the TFC-PIP-blank membrane [43].

**Figure 2:**
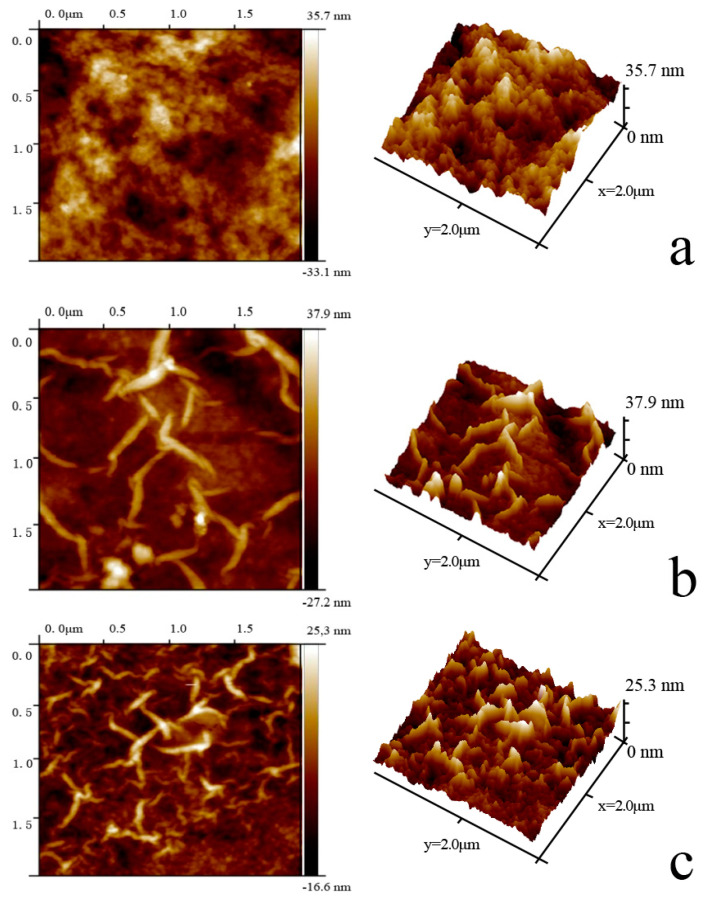
AFM surface topography of TFC-PEI-blank (**a**), TFN-PEI-GO-20 (**b**), TFN-PEI-GO-40 (**c**).

The surface roughness of the fabricated NF membranes was further characterized by AFM. The key parameters of surface roughness include the mean roughness (*Ra*), the root mean square of Z data (*Rq*), and the mean difference between the five highest peaks and five lowest valleys (*Rz*). According to Figure 2 and Table 3, the surface roughness of the TFN-PEI-GO membranes was dramatically smaller than the TFC-PIP-blank and TFN-PIP membranes in our previous reports [43], which was consistent with the SEM results. Meanwhile, the surface roughness of the TFN-PEI-GO membranes gradually decreased with the increase of GO concentration, which was also ascribed to the reduced diffusion rate of PEI inhibited by the steric hindrances and hydrogen bonding interactions from GO on PEI. The retarded IP process led to the formation of a smoother active layer. In addition, it was found that the introduction of GO resulted in the formation of the wrinkled and patterned surfaces, which was probably caused by the reduced diffusion rate of PEI monomers. This result was similar to the findings from Zhu’s research group [49]. However, they thought that the wrinkled and patterned membrane surfaces derived from the wrinkled GO nanosheets with the appropriate lateral size as the templates [49]. With the increase of the GO concentration, more and more wrinkled and patterned structures appeared on membrane surfaces, which was expected to increase the effective filtration area and, accordingly, improve membrane flux.

### 3.2. Membrane Surface Properties

**Figure 3:**
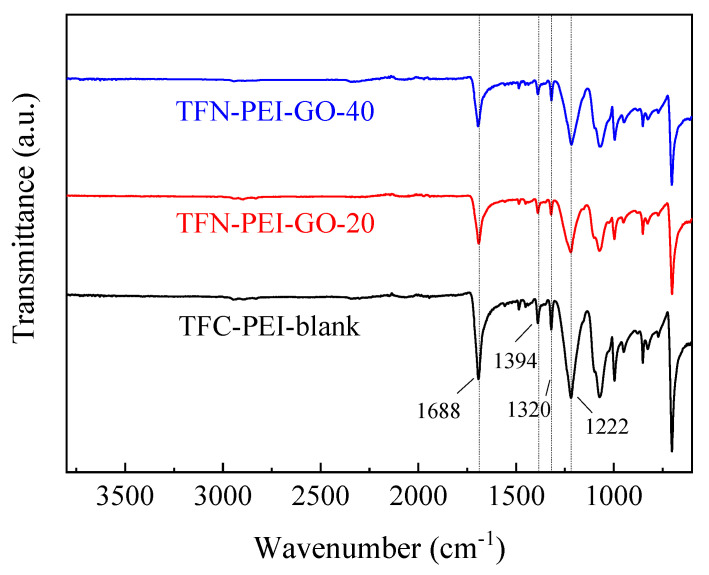
ATR-FTIR spectra of composite NF membranes.

The surface chemical groups of the fabricated NF membranes were characterized by ATR-FTIR. According to Figure 3, the characteristic peaks at 1688 and 1394 cm^−1^ were assigned to the typical C=O stretching vibrations of the amide-I band and the stretching vibration of C–N in the amide [43,50], respectively. Meanwhile, the peaks at around 1222 cm^−1^ and 1320 cm^−1^ were assigned to the bending vibration of N–H [51]. This indicated the successful formation of the polyamide active layers on the PSU support. Additionally, the absorption peak intensities of the TFN-PEI-GO membranes were slightly lower than that of the TFC-PEI-blank membrane, which was probably due to the embedded GO nanosheets and the relatively thinner active layer.

**Figure 4:**
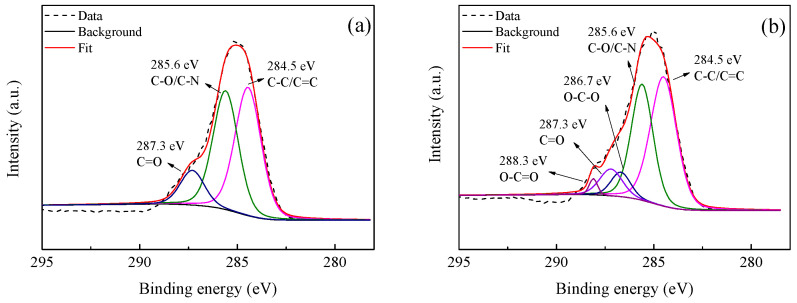

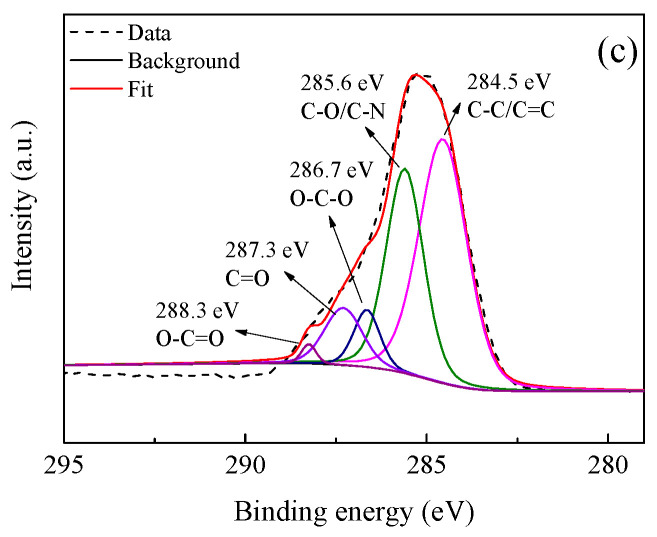
XPS C1s spectra of TFC-PEI-blank (**a**), TFN-PEI-GO-20 (**b**)**,** and TFN-PEI-GO-40 (**c**).

XPS had higher detective sensitivity than ATR-FTIR, which was further employed to analyze the chemical compositions of the membrane surfaces. As shown in Figure 4, the peaks at 284.5, 285.6, and 287.3 eV were assigned to C–C/C=C, C–O/C–N, and C=O functional groups [52], respectively, which also suggested that the polyamide active layer was successfully generated after IP process. Moreover, two new peaks at 286.7 and 288.3 eV attributed to C–O–C and O–C=O were found for the TFN-PEI-GO-20 and TFN-PEI-GO-40 membranes. These epoxy and carboxyl groups derived from GO, indicating the successful incorporation of GO into the active layer after the IP process.

**Figure 5:**
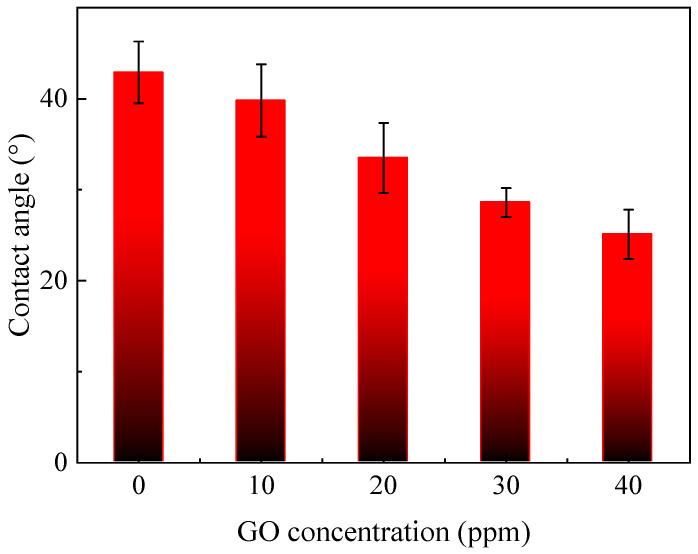
Effects of GO concentrations on water contact angle (WCA) of NF membranes.

The water contact angle (WCA) is commonly used to evaluate membrane hydrophilicity. The relationship between the GO concentration and the WCA of the composite NF membranes is depicted in Figure 5. With the increase of the GO concentration, the WCA of the TFN-PEI-GO membranes gradually decreased. This indicated that the membrane hydrophilicity was enhanced with the increase of GO concentration. GO nanosheets contain a large number of oxygen-containing groups such as hydroxyl and carboxyl groups, which contributed to improving the membrane hydrophilicity. When the GO concentration increased from 0 to 40 ppm, the WCA of the fabricated NF membranes significantly decreased from 42.0° to 25.1°.

### 3.3. Water Flux and Salt Rejection

**Figure 6:**
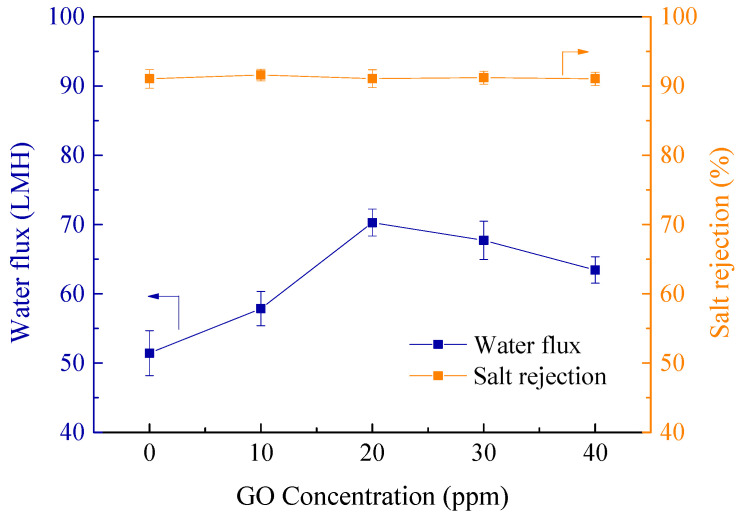
Effects of GO concentrations on separation performance of membranes.

The water fluxes and salt rejections of composite NF membranes were measured and are shown in Figure 6. Notably, there was a critical GO concentration for water flux. With the addition of 20 ppm GO into aqueous solution, the TFN-PEI-GO-20 membrane exhibited the highest water flux of 70.3 LMH, which was 36.8% higher than the TFC-PEI-blank membrane. However, the water flux turned to decrease when the GO concentration was beyond the critical concentration. It was speculated that the GO agglomeration at high concentration led to the increased membrane filtration resistance and the decreased flux. With the help of DMAP catalyst, the TFN-PEI-GO membranes demonstrated the relatively high and stable Na_2_SO_4_ rejections, above 91%, regardless of the variation of GO concentration. This suggested that the introduction of GO improved water flux without sacrificing the salt rejection. The increase of water flux benefitted from: (1) The introduction of GO generated a thinner active layer, which correspondingly alleviated the membrane filtration resistance; (2) the introduction of GO led to the formation of the wrinkled membrane surface, which increased the effective membrane filtration area; (3) the hydrophilicity of the active layer was enhanced with embedding GO; and (4) GO provided additional transmission channels with less mass transfer resistance to water molecules.

**Figure 7:**
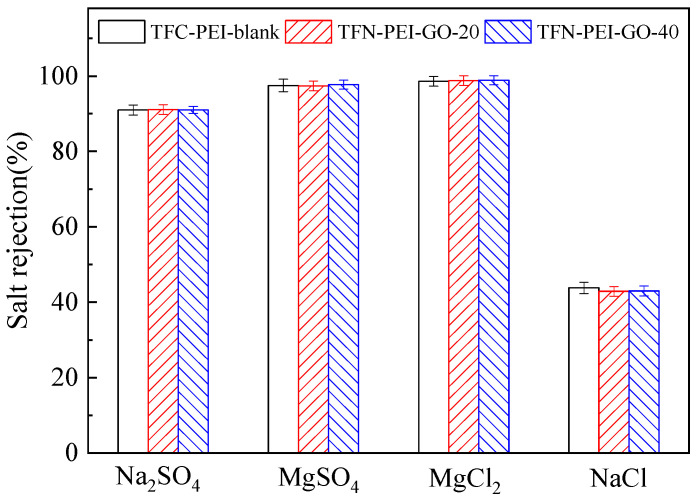
Different salt rejections of the selected NF membranes.

In order to explore in depth the separation mechanisms of the positively charged PEI-based NF membranes, the rejections of four kinds of salts were measured and are illustrated in Figure 7. The mass concentration (500 ppm) of different salts was equal and relatively low in this study. Thus, the influence of molar concentration variation on the salt rejection could be ignored for the dilute salt solutions. Because macromolecular PEI is a cationic polyelectrolyte with branched chains, containing a large number of primary amine groups, secondary amine groups, and tertiary amine groups, the fabricated PEI-based NF membranes presented the positively charged surfaces. The order of salt rejections was MgCl_2_ ≈ MgSO_4_ > Na_2_SO_4_ > NaCl, which matched very well with the characteristics of the positively charged NF membrane [33]. This order was completely different from the negatively charged PIP-based NF membranes. The salt rejections of the TFN-PEI-GO membranes were similar to those of the TFC-PEI-blank membrane, suggesting that the loading of GO at a low concentration did not change the characteristics of the TFN-PEI-GO membranes.

It is well known that the NF separation mechanisms include the Donnan exclusion effect and the size exclusion effect. Therefore, the positively charged TFN-PEI-GO membranes generated the stronger electrostatic repulsion toward the divalent Mg^2+^ than the monovalent Na^+^. As a result, the rejection of divalent Mg^2+^ was greater than that of monovalent Na^+^. For the salts having the same divalent Mg^2+^, the positively charged TFN-PEI-GO membranes also produced the stronger electrostatic attraction toward the divalent SO_4_^2−^ than the monovalent Cl^−^. The strong adsorption of SO_4_^2−^ significantly weakened the positively charged properties of membrane surfaces. As a result, the rejection of MgSO_4_ was lower than that of MgCl_2_. However, for the salts having the same monovalent Na^+^, the Na_2_SO_4_ rejection was much higher than NaCl rejection because SO_4_^2−^ has a greater hydration radius than Cl^−^, which indicated that the size exclusion effect was more dominant than the Donnan exclusion effect.

### 3.4. Removal of Heavy Metals

**Figure 8:**
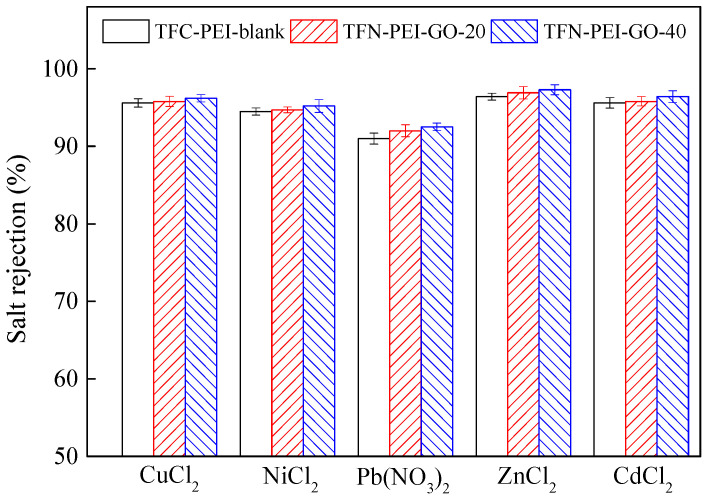
Rejections of different heavy metal salt solutions.

The TFC-PEI-blank, TFN-PEI-GO-20, and TFN-PEI-GO-40 were selected to investigate their separation performance of removing heavy metals. According to Figure 8, the selected NF membranes showed high rejections, above 91%, toward five kinds of heavy metal salts. The rejection of Pb(NO_3_)_2_ was 91% and the rejections of the other heavy metal salts ranged from 94% to 97%. This indicated that the positively charged NF membranes fabricated from PEI monomers were helpful to enhance the removals of heavy metals. Because both the TFC-PEI-blank membrane and the TFN-PEI-GO membranes were positively charged, these membranes presented the same sequence for removing heavy metals: ZnCl_2_ > CdCl_2_ ≈ CuCl_2_ > NiCl_2_ > Pb(NO_3_)_2_. This result was consistent with the hydration radius sequence of various heavy metal ions, as listed in Table 2: Zn^2+^ > Cd^2+^ > Cu^2+^ > Ni^2+^ > Pb^2+^. Five kinds of heavy metal ions belong to divalent cations. Thus, the rejections depended on the hydration radius based on size exclusion effect. The larger hydration radius of heavy metal ion contributed to the high rejection of heavy metal ion.

**Figure 9:**
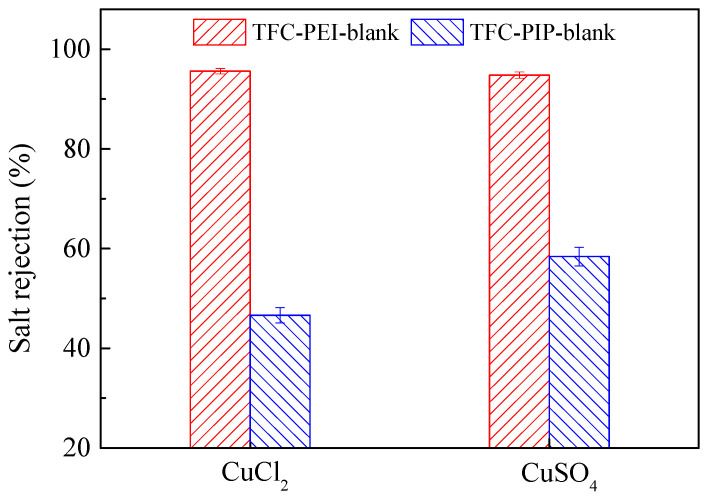
Cu^2+^ rejection of different composite NF membranes.

The influences of different aqueous reactive monomers (PIP vs. PEI) on the copper removal of the TFC NF membranes were further investigated. As shown in Figure 9, regardless of CuSO_4_ or CuCl_2_, the Cu^2+^ rejection of the positively charged TFC-PEI-blank membrane was remarkably higher than that of the negatively charged TFC-PIP-blank membrane. For the TFC-PEI-blank membrane, the rejections of both CuSO_4_ and CuCl_2_ were above 94%, and the CuSO_4_ rejection was slightly lower than the CuCl_2_ rejection because the positively charged TFC-PEI-blank membrane generated the stronger electrostatic attraction toward the divalent SO_4_^2−^ than the monovalent Cl^−^. This suggested that the Donnan exclusion effect was predominant compared with the size exclusion effect. On the contrary, the negatively charged TFC-PIP-blank membrane presented the stronger electrostatic repulsion toward the divalent SO_4_^2−^ than the monovalent Cl^−^. Thus, both the Donnan exclusion and size exclusion preferred to increase the CuSO_4_ rejection rather than the CuCl_2_ rejection for the TFC-PIP-blank membrane.

### 3.5. Antifouling Performance

**Figure 10:**
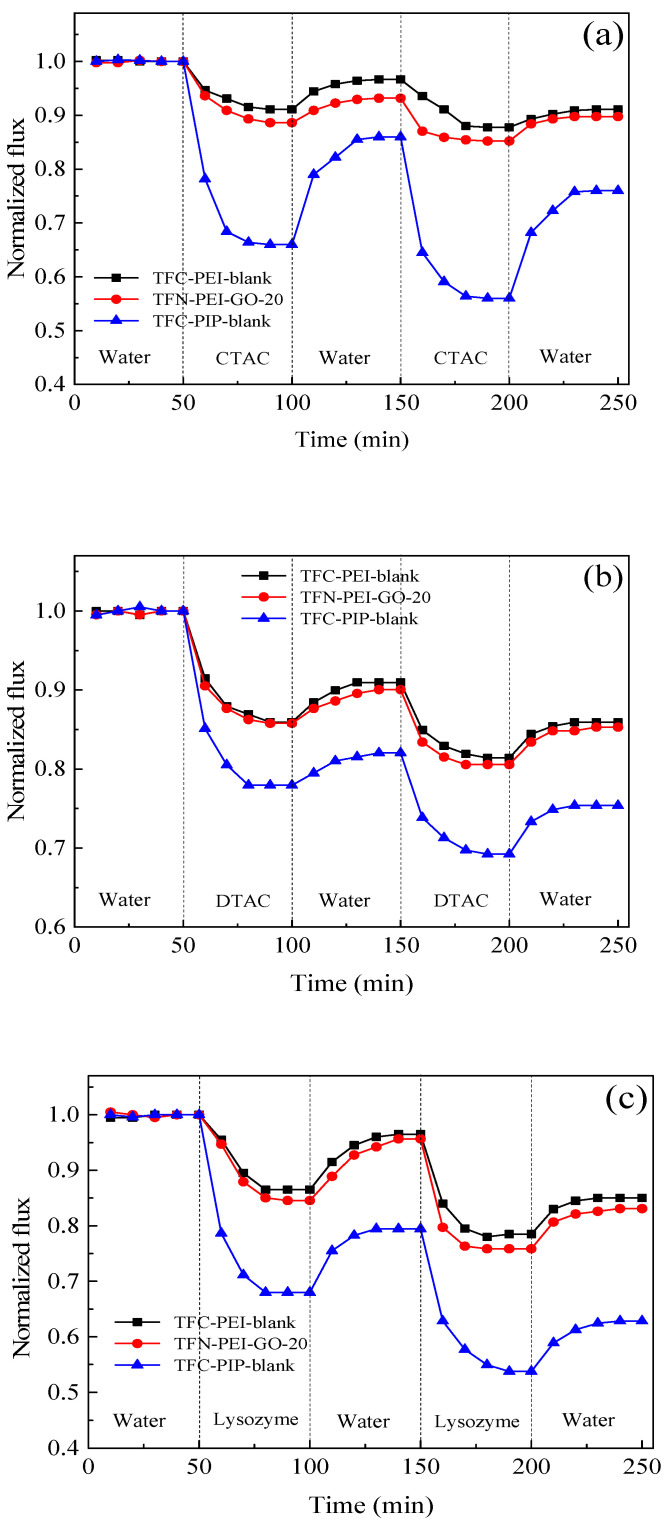
Antifouling performance of composite NF membranes using different foulants: CTAC (**a**), DTAC (**b**)**,** and lysozyme (**c**).

Two kinds of cationic surfactant (CTAC and DTAC) and lysozyme (LYZ) were used to evaluate the antifouling performance of the fabricated NF membranes. According to Figure 10, regardless of CTAC, DTAC, or LYZ, the normalized water fluxes of the TFC-PEI-blank and TFN-PEI-GO-20 membranes were significantly higher than those of the TFC-PIP-blank membrane.

After fouling by CTAC and water washing, the normalized water fluxes of the TFC-PEI-blank and TFN-PEI-GO-20 membranes reached 91.1% and 89.7%, respectively. However, the normalized water flux of the TFC-PIP-blank membrane was only 76%. After fouling by DTAC, the normalized water fluxes of the TFC-PEI-blank and TFN-PEI-GO-20 membranes reached 85.9% and 85.3%, respectively. However, the normalized water flux of TFC-PIP-blank was only 75.4%. LYZ is positively charged under neutral testing conditions due to its isoelectric point of 10.7. After fouling by LYZ, the normalized water fluxes of the TFC-PEI-blank and TFN-PEI-GO-20 membranes reached 85.0% and 83.1%, respectively, while the normalized water flux of the TFC-PIP-blank membrane was only 62.8%. Therefore, for the positively charged foulants, the above results indicated that the positively charged TFC-PEI-blank and TFN-PEI-GO-20 membranes had superior antifouling performance over the negatively charged TFC-PIP-blank membrane.

On the one hand, both the TFC-PEI-blank and TFN-PEI-GO-20 membranes possessed a more hydrophilic and smoother surface than the TFC-PIP-blank membrane, which was helpful to reduce membrane fouling. On the other hand, the positively charged TFC-PEI-blank and TFN-PEI-GO-20 membranes generated the stronger electrostatic repulsion toward the cationic surfactants and the positively charged LYZ, which further alleviated the membrane fouling. On the contrary, the negatively charged TFC-PIP-blank membrane had the strong electrostatic attraction toward the cationic surfactants and the positively charged LYZ, which resulted in the severe membrane fouling.

Although the TFN-PEI-GO-20 membrane was more hydrophilic compared with the TFC-PEI-blank membrane, the TFN-PEI-GO-20 membrane presented a slightly more severe membrane fouling than the TFC-PEI-blank membrane. This was caused by the higher flux of the TFN-PEI-GO-20 membrane than that of the TFC-PEI-blank membrane. It means that more foulants were adsorbed on the membrane surface and the concentration polarization was intensified due to the high membrane flux, which would aggravate membrane fouling.

### 3.6. Chlorine Resistance

In view of the degradation mechanism of the PA TFC membranes, the PEI-based PA membranes are vulnerable to chlorine attack due to a large amount of end amine groups for N-chlorination [35]. Therefore, an important objective of the present study was to enhance the chlorine resistance of the PEI-based PA membranes by incorporating GO nanosheets into the active layer.

**Figure 11:**
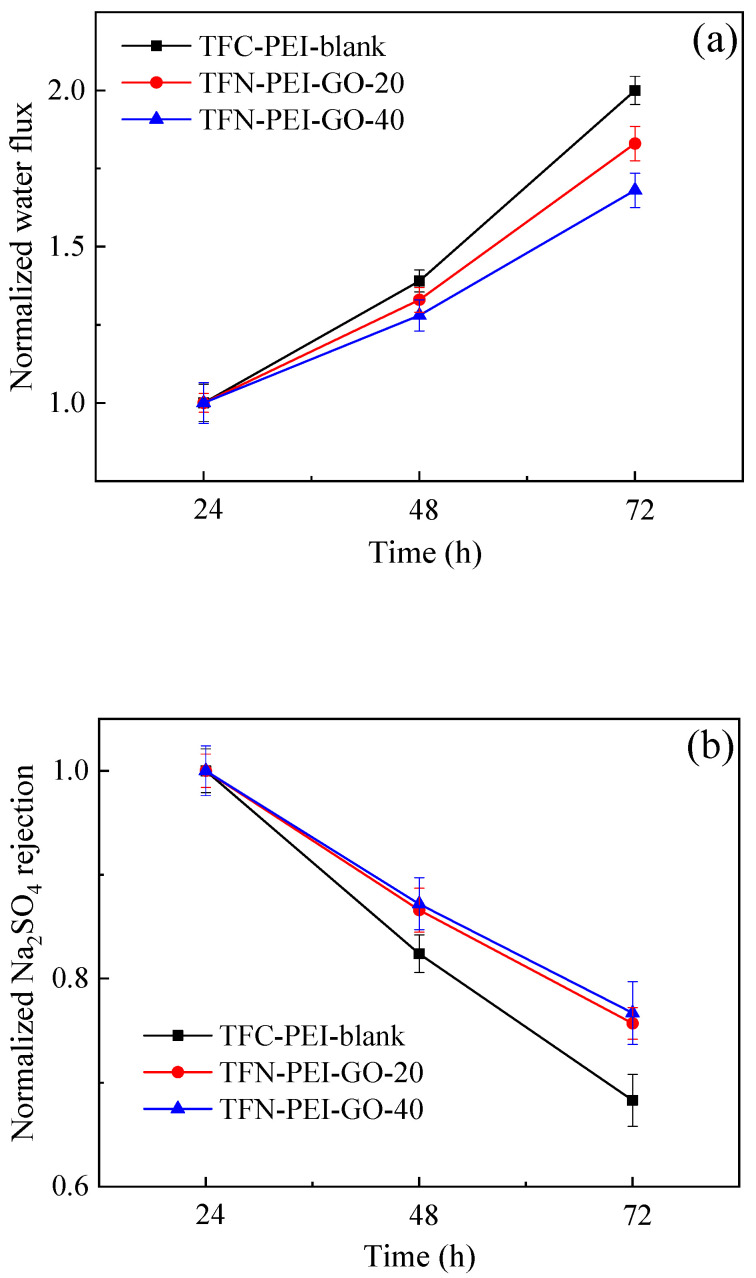
Effects of the chlorine exposure time on the normalized water flux (**a**) and Na_2_SO_4_ rejection (**b**) of the composite NF membranes.

The normalized water flux and the normalized Na_2_SO_4_ rejection were used to evaluate the chlorine resistances of the fabricated NF membranes. As shown in Figure 11, the normalized water flux significantly increased with the increasing chlorine exposure time, and the variation of the normalized water flux was TFC-PEI-blank > TFN-PEI-GO-20 > TFN-PEI-GO-40. Meanwhile, the normalized Na_2_SO_4_ rejection decreased with the increasing chlorine exposure time and displayed the opposite order: TFC-PEI-blank < TFN-PEI-GO-20 < TFN-PEI-GO-40. These results indicated that the incorporation of GO in the active layer contributed to improving the chlorine resistance of the PEI-based PA NF membranes. On the one hand, the skeleton structure of GO could absorb chlorine radicals to form O–Cl bonds, accordingly reducing the chlorine radical attack toward the PA layer [53]. On the other hand, the embedded GO nanosheets provided additional protection for the underlying PA, ascribed to their large specific surface area and the hydrogen bonding between GO and PA [54]. Thus, the chlorine resistance of the PEI-based PA NF membrane increased with the increase of GO concentration.

## 4. Conclusions

To construct the positively charged NF membrane with enhanced performance, the PEI-based TFN NF membrane using PEI as the aqueous reactive monomers was fabricated by embedding GO into the active layer via the IP process. After incorporating an appropriate amount of GO into the PEI-based PA active layer, the optimal TFN-PEI-GO-20 membrane showed the maximal water flux of 70.3 L·m^−2^·h^−1^ without sacrificing salt rejection, which was 36.8% higher than the TFC-PEI-blank membrane. Meanwhile, the positively charged TFC-PEI-blank and TFN-PEI-GO membranes presented superior rejections for heavy metal ions (Zn^2+^, Cd^2+^, Cu^2+^, Ni^2+^, and Pb^2+^) due to the synergistic effects of Donnan exclusion and size exclusion. Furthermore, both the TFC-PEI-blank and TFN-PEI-GO-20 membranes exhibited excellent antifouling performance toward the cationic surfactants and basic protein (LYZ), owing to the hydrophilic, smooth, and positively charged surface. Finally, the TFN-PEI-GO membranes presented the enhanced chlorine resistances due to the extra protection from GO. Therefore, the positively charged PEI-based TFN NF membrane embedded with GO shows a promising potential for wastewater purification from heavy metal ions.

## Figures and Tables

**Table 1:** Preparation conditions of composite NF membranes.

Membrane ID	PEI (wt%)	TMC (w/v%)	TEA (wt%)	DMAP (wt%)	GO (ppm)
TFC-PEI-blank	1	0.2	2	0.08	0
TFN-PEI-GO-10	1	0.2	2	0.08	10
TFN-PEI-GO-20	1	0.2	2	0.08	20
TFN-PEI-GO-30	1	0.2	2	0.08	30
TFN-PEI-GO-40	1	0.2	2	0.08	40

**Table 2:** Key parameters of the related ions [46].

Ions	Hydrated Radius (nm)
Na^+^	0.358
Mg^2+^	0.428
Cl^−^	0.332
SO_4_^2−^	0.379
Zn^2+^	0.430
Cd^2+^	0.426
Cu^2+^	0.419
Ni^2+^	0.404
Pb^2+^	0.401
NO_3_^−^	0.335

**Table 3:** Roughness of the fabricated NF membranes.

Membrane ID	Roughness (nm)		
	*R_a_*	*R_q_*	*R_z_*
TFC-PEI-blank	7.81	9.90	7.57
TFN-PEI-GO-20	6.99	9.12	14.6
TFN-PEI-GO-40	4.41	5.81	8.01
